# Characteristic MR Signal of Velopharyngeal-Related Muscles on Cine-MRI in Patients with Oral Cancers and Preserved Swallowing Function: An Exploratory Feasibility Study

**DOI:** 10.3390/tomography12080117

**Published:** 2026-08-20

**Authors:** Shun Nishimura, Masafumi Oda, Mana Hayakawa, Shirou Tabe, Hiroki Tsurushima, Susumu Nishina, Kozue Iwawaki-Otsuka, Nao Wakasugi-Sato, Shinobu Matsumoto-Takeda, Osamu Takahashi, Kazuya Haraguchi, Shinji Yoshii, Manabu Habu, Izumi Yoshioka, Toyohiro Kagawa, Yasuhiro Morimoto

**Affiliations:** 1 Division of Oral and Maxillofacial Radiology, Kyushu Dental University, Kitakyushu 803-8580, Japan; nishimura.sn@fdcnet.ac.jp (S.N.); r07oda@fa.kyu-dent.ac.jp (M.O.); r22nishina@fa.kyu-dent.ac.jp (S.N.); r24iwawaki@fa.kyu-dent.ac.jp (K.I.-O.); r16wakasugi@fa.kyu-dent.ac.jp (N.W.-S.); r17matsumoto@fa.kyu-dent.ac.jp (S.M.-T.); 2 Section of Oral and Maxillofacial Image Diagnosis, Department of Diagnostics and General Care, Fukuoka Dental College, Fukuoka 814-0175, Japan; kagawat1@fdcnet.ac.jp; 3 Division of Maxillofacial Surgery, Kyushu Dental University, Kitakyushu 803-8580, Japan; r23hayakawa@fa.kyu-dent.ac.jp (M.H.); r13tabe@fa.kyu-dent.ac.jp (S.T.); r07takahashi@fa.kyu-dent.ac.jp (O.T.); r11haraguchi@fa.kyu-dent.ac.jp (K.H.); h-manabu@kyu-dent.ac.jp (M.H.); 4 Division of Oral Medicine, Kyushu Dental University, Kitakyushu 803-8580, Japan; r17tsurushima@fa.kyu-dent.ac.jp (H.T.); r13yoshioka@fa.kyu-dent.ac.jp (I.Y.); 5 Division of Learning Design, Kyushu Dental University, Kitakyushu 803-8580, Japan; r08yoshii@fa.kyu-dent.ac.jp

**Keywords:** cine-MRI, velopharyngeal, swallowing, dynamic, function, oral cancer, characteristic MR signal

## Abstract

Background/Objectives: Surgically treated oral cancer patients often face velopharyngeal insufficiency, which adversely impacts their quality of life (QOL). Our past investigations revealed noticeable MR signal shifts in velopharyngeal-related muscles during water deglutition on CMR among healthy individuals. We hypothesized that analyzing these distinct signal fluctuations could offer a novel visual perspective for detecting deglutition anomalies in surgical oral cancer cases. This feasibility study aimed to assess velopharyngeal-related muscular performance via CMR in post-operative oral cancer patients. Methods: Nineteen patients with oral malignancies were prospectively evaluated pre- and post-operatively. Twelve quantitative CMR metrics alongside subjective dysphagia scores were investigated. We analyzed whether signal intensity (SI) ratio alterations in these muscles correlated with dysphagia severity and clinical parameters. Results: Although three specific CMR parameters significantly worsened post-operatively, muscle MR signal values increased during deglutition without significant variation between pre- and post-surgical states. Subjective swallowing assessments remained entirely normal across all subjects. Conclusions: This preliminary investigation indicates that CMR can visualize stable velopharyngeal-related muscle activity after surgery in oral cancer patients maintaining adequate swallowing status. Nevertheless, confirmation via larger, symptomatic patient cohorts alongside gold-standard techniques remains mandatory.

## Simple Summary

Cine-magnetic resonance imaging (CMR) holds potential for demonstrating that velopharyngeal-related muscular activity remains stable after surgical intervention in oral cancer subjects who retain adequate deglutition ability.

## 1. Introduction

The oral cavity plays a vital role through a complex series of movements during biting, swallowing, and speech. Oral cancers and their treatments may cause severe functional limitations, including swallowing-related disorders such as aspiration pneumonia [1,2,3,4,5]. For both doctors and patients, it is necessary to establish safe, precise, and objective imaging techniques for the evaluation of swallowing function. In our previous studies, we reported the use of cine-magnetic resonance imaging (CMR) for evaluating swallowing function and therapeutic response in patients with oral cancers, and demonstrated that CMR can directly visualize swallowing dynamics and objectively evaluate swallowing complaints in these patients [6,7,8].

Velopharyngeal insufficiency may lead to a decreased quality of life (QOL) in patients undergoing surgery for oral cancers because of problems such as aspiration, dysphonia, impaired fluid intake, and stridor [1,9,10]. The development of precise treatments for velopharyngeal insufficiency is therefore an important clinical and social issue. We previously demonstrated that the MR signals of velopharyngeal-related muscles are markedly altered during water swallowing on CMR in volunteers [11].

Therefore, we hypothesized that the characteristic MR signal change in the velopharyngeal-related muscles observed on CMR could enable visualization of swallowing anomalies in patients with oral cancers and their treatments from a novel perspective. We further hypothesized that changes in the signal intensity (SI) ratio of these muscles would correlate with dysphagia severity and clinical parameters. While our previous studies established the basic feasibility of dynamic cine-MRI in healthy volunteers and general oral cancer cohorts [6,7,8,11], the present study specifically focuses on quantifying the signal intensity changes in velopharyngeal-related muscles in surgical oral cancer patients before and after operation, evaluating its exploratory correlation with surgical invasiveness and objective transit parameters.

In the present study, we performed pre- and post-operative evaluations of the relationship between the MR signal of the velopharyngeal-related muscles during water swallowing and self-reported dysphagia scores reflecting swallowing status, 12 CMR-related parameters, T classification (tumor stage) in pre-operative patients, and the degree of surgical invasiveness in patients with oral cancers.

The present study involves a patient population that is entirely different from that of our three previously published work, and the figures are also entirely different. Regarding the patient cohort, Tanaka et al. evaluated the swallowing status of oral cancer patients before and after surgery using cine-MRI parameters [6]. Nishimura et al. focused specifically on patients with tongue cancer, analyzing pre- and post-operative changes using cine-MRI and demonstrating changes in parameters following improvements in swallowing function [7]. At the time these two papers were published, Joujima et al. had not yet confirmed that cine-MRI could evaluate changes in signal intensity in muscles involved in swallowing and mastication during speech and water-drinking [11]. The present study used cine-MRI to evaluate water drinking in oral cancer patients before and after surgery—a method previously employed by Joujima et al. to assess healthy subjects—and evaluated signal changes in muscles involved in swallowing [11]. Therefore, unlike any of the previous patient cohorts, all data were newly acquired. As noted at the beginning of the present study, the fact that we were able to perform this functional assessment in oral cancer patients demonstrates the novelty of this work, and we consider it to be of great significance.

## 2. Materials and Methods

### 2.1. Subjects

Nineteen consecutive oral cancer patients (6 males, 13 females; mean age: 64.2 years; range: 45–84 years) treated at Kyushu Dental University Hospital (2024–2025) were prospectively enrolled in the feasible present study. Histopathological confirmation was documented for all cases. Primary lesion distribution included the tongue, lower gingiva, upper gingiva, buccal mucosa, palate, and lip. Approval was granted by the Institutional Review Board of Kyushu Dental University (No. 20-56), with written informed consent obtained from each participant.

Pre-operative T classifications comprised: Tis (n = 3), T1 (n = 5), T2 (n = 4), T3 (n = 2), and T4 (n = 5). Tumor sites included tongue (n = 12), mandibular gingiva (n = 5), maxillary gingiva (n = 1), and floor of mouth (n = 1).

Surgical procedures were categorized into four invasiveness levels per Tanaka et al. [7,8,12,13]: Type I (local excision of soft tissues or marginal rim resections); Type II (marginal resections combined with soft tissue flap reconstruction or mandibular segmental resections); Type III (major glossectomies with reconstruction or mandibular transections); and Type IV (neck dissection performed alongside any of the aforementioned procedures).

### 2.2. Imaging Parameters for CMR

Scans were conducted using a 1.5-T scanner (EXCELART VantageTM powered by Atlas; Toshiba, Tokyo, Japan) equipped with a circular neck array coil to capture movements of the occipital muscles (OMs), soft palate (SP), masseter muscles (MMs), levator veli palatini muscles (LVPMs), and superior pharyngeal constrictor muscles (SPCMs). OMs served as reference tissue due to their functional inactivity during deglutition. CMR protocols adapted the methodology of Joujima et al. [11]. Static multiplanar T1- and T2-weighted images preceded dynamic imaging for anatomical localization and structural screening. Scans required a supine posture, altering gravity-driven bolus motion relative to upright deglutition. Continuous radiofrequency-spoiled steady-state free precession (SSFP) radial field-echo sequences were acquired (Table 1: TR = 3.2 ms; TE = 1.6 ms; FA = 45°; FOV = 250 × 225 mm^2^; slice thickness = 8 mm). Ten radial spokes sampled k-space, achieving a temporal frame rate of 28 frames/s (~36 ms acquisition time per frame) via sliding-window reconstruction interpolation.

Following routine structural MRI, 12 dynamic CMR parameter sets were obtained according to modified protocols [6,7]. Scans utilized T2-weighted dynamics to track water bolus motion safely while minimizing aspiration risk. Mid-sagittal CMR sequences captured 5 mL saline swallowing across the oral, nasopharyngeal, and pharyngeal regions [6,11]. Nasopharyngeal seal dynamic imaging was performed across axial planes (at SP level) and oblique coronal planes (aligned with LVPMs) [11,14,15].

A dentist stationed by the scanner provided verbal cues to synchronize swallowing initialization with scan execution. Single-swallow runs lasted approximately 10 s per acquisition plane. Ideal anatomical movement planes during resting and active phases were selected for analysis.

### 2.3. Image Evaluation Analysis

Images were evaluated independently by a certified oral radiologist (S.N.) blinded to patient clinical records, following established criteria [8,11].

### 2.4. Self-Reported Dysphagia Scores

Patients completed standardized self-report surveys detailing perceived deglutition status pre- and post-operatively, categorized as normal, mildly impaired, or severely impaired [6,7].

### 2.5. Evaluations of Velopharyngeal-Related Muscles on CMR

**Figure 1:**
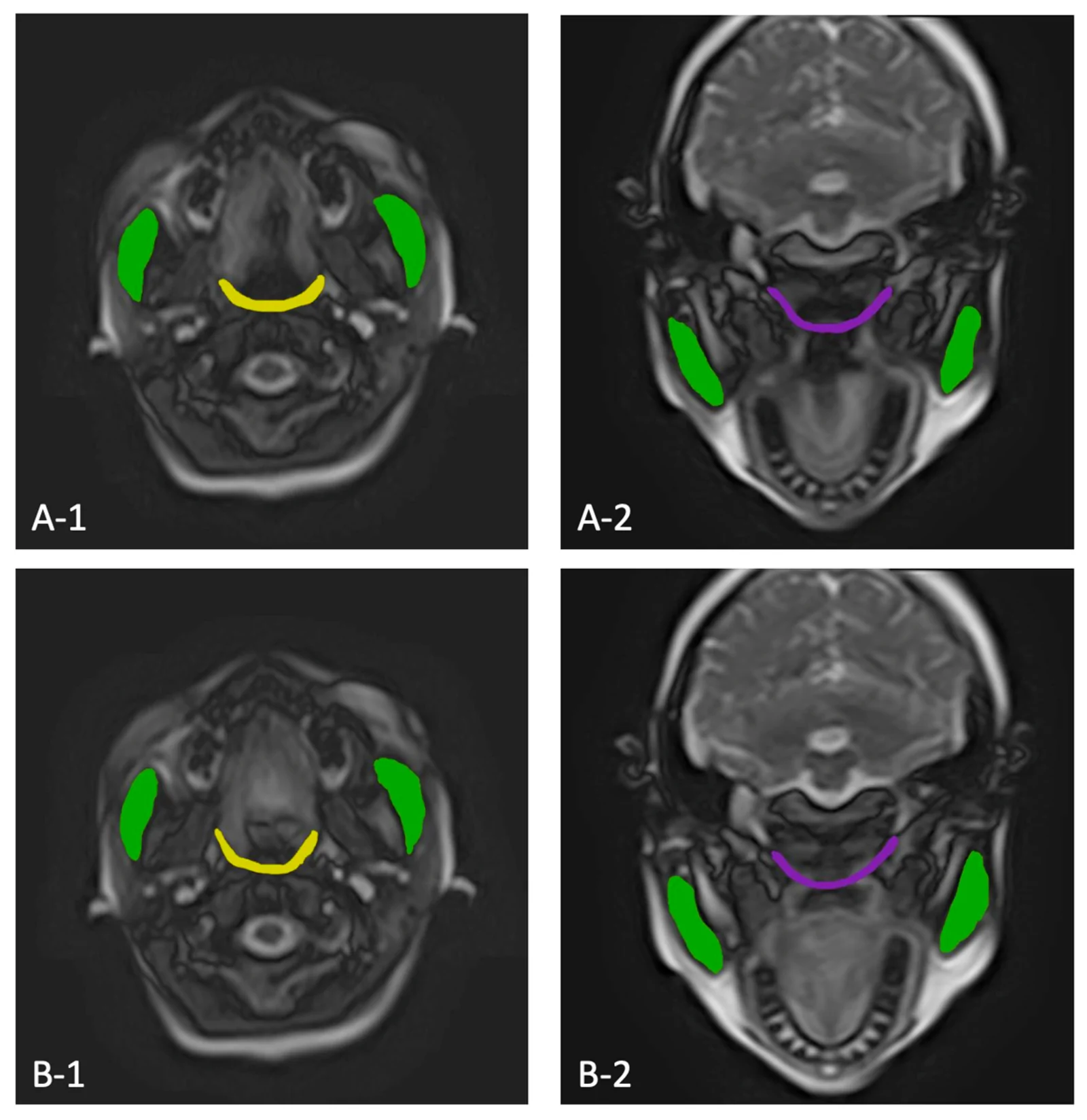
The signal intensities were calculated on cine-MRI during rest (**A**) and swallowing (**B**). The signal intensities of the LVPMs (purple), SPCMs (yellow), and MMs (green) were measured on axial images (**A-1**,**B-1**) and oblique coronal images (**A-2**,**B-2**).

Signal intensity fluctuations in OMs, SP, MMs, LVPMs, and SPCMs were calculated between resting and water deglutition phases [11,16]. Bilateral regions of interest (ROIs) were positioned on resting scans and translated to corresponding dynamic deglutition frames (Figure 1). OMs served as an internal baseline control. Muscular activation ratios were computed as SIworking/SIrest.

### 2.6. Evaluation of Swallowing Functions on CMR

Dynamic swallowing performance was categorized by structural execution, temporal metrics, and clearance efficiency. Timing variables followed Zhang et al. [17] based on Logemann’s six-valve framework [18,19,20,21,22]. Valve 1 remained closed throughout. Valve 2 tracked orovelar opening time (OOT), Valve 3 marked velopharyngeal closure time via first Passavant ridge appearance (PR1), Valve 4 recorded glottal closure time (GCT), Valve 5 registered second Passavant ridge appearance (PR2), and Valve 6 denoted esophageal opening time (EOT). Laryngeal ascent time (LAT), laryngeal descent time (LDT), and vallecular/pyriform sinus stasis durations were recorded.

A tissue immobility index (range: 9 to 23) was adapted from Kreeft et al. [23] to evaluate mobility across five anatomical regions (anterior tongue, tongue base, posterior pharyngeal wall, palate, floor of mouth; 1 = normal to 3 = immobile) and inter-structural contact points across four junctions (1 = normal contact, 2 = absent contact). Non-evaluable elements received baseline scores of 1.

### 2.7. Statistical Analysis

Statistical procedures were conducted using SPSS v23 (SPSS Inc., Chicago, IL, USA). ANOVA, Spearman rank tests, and paired/unpaired Student’s *t*-tests were executed where appropriate. Statistical significance was set at *p* < 0.05. Multiple testing corrections applied Benjamini–Hochberg false discovery rate (FDR) adjustments (q-values). Cohen’s dz/d and partial eta squared (η^2^) quantified effect sizes. Inter-rater reliability was measured using Kappa (κ) statistics.

## 3. Results

### 3.1. Correlation Between Pre-Operative SI_working_/SI_rest_ and Dysphagia Score, T Classification, Tumor Site, and Surgical Procedure

**Figure 2:**
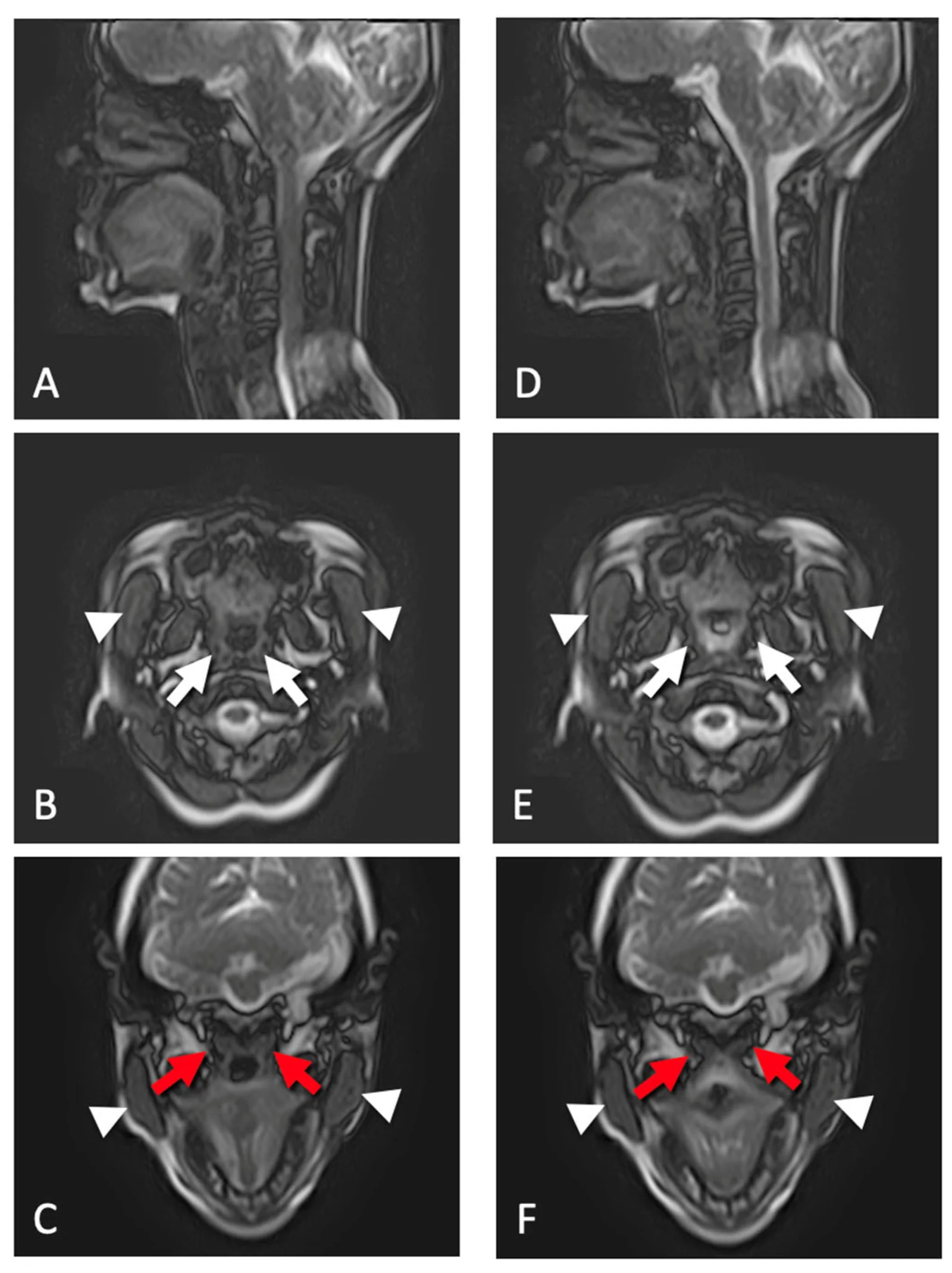
(**A**), Mid-sagittal; (**B**), axial; and (**C**), oblique coronal images of the velopharyngeal area during rest (1) and water swallowing (2). During water swallowing, velopharyngeal closure is visualized, with the SP and LVPMs moving backward to contact the posterior wall of the oropharynx anteriorly (**A**–**C**), and moving backward to contact the posterior wall of the oropharynx (**D**–**F**). In addition, the SPCMs expand anteriorly and bilaterally, resulting in posterior, right-, and left-sided closure (**D**–**F**). The signals of the SPCMs ((**B**,**E**); white arrows) and LVPMs ((**C**,**F**); red arrows) are conspicuously increased compared with rest. The signal intensities of the MMs ((**B**,**C**,**E**,**F**); arrowheads) show little change between rest and water swallowing.

The flow direction to the esophagus could be judged on pre-operative CMR in all 19 patients (Figure 2, Video S1), but the flow direction to the trachea could not be judged in the feasible present study. The MR signals of the LVPMs and SPCMs significantly increased compared with those of the OMs, SP, and MMs in all 19 patients during water swallowing (Figure 2, Video S1, Table 2). All self-assessments regarding water swallowing were normal.

Video S1. Video obtained during water swallowing shows the oral cavity, mid-velopharyngeal, and esophageal areas (mid-sagittal) in the same patient as in Figure 2. During water swallowing, the signals of the LVPMs and SPCMs are conspicuously increased compared with rest. The signal intensities of the MMs show little change between rest and water swallowing. At the same time, the velopharyngeal closure is appropriately visualized, with the SP and LVPMs moving backward to contact the posterior wall of the oropharynx.

There was no significant correlation between the SIworking/SIrest of the respective velopharyngeal-related muscles and the self-reported dysphagia scores at either time point, pre-operative T classification (Table 3), tumor site (Table 4), or surgical procedure (Table 5). However, regarding comparisons by tumor site, since each group included only one patient, it was not possible to perform a statistically significant comparison; therefore, the results are presented descriptively.

### 3.2. Correlation Between Changes in SI_working_/SI_rest_ and CMR Parameters Between Pre- and Post-Operative Assessments

**Figure 3:**
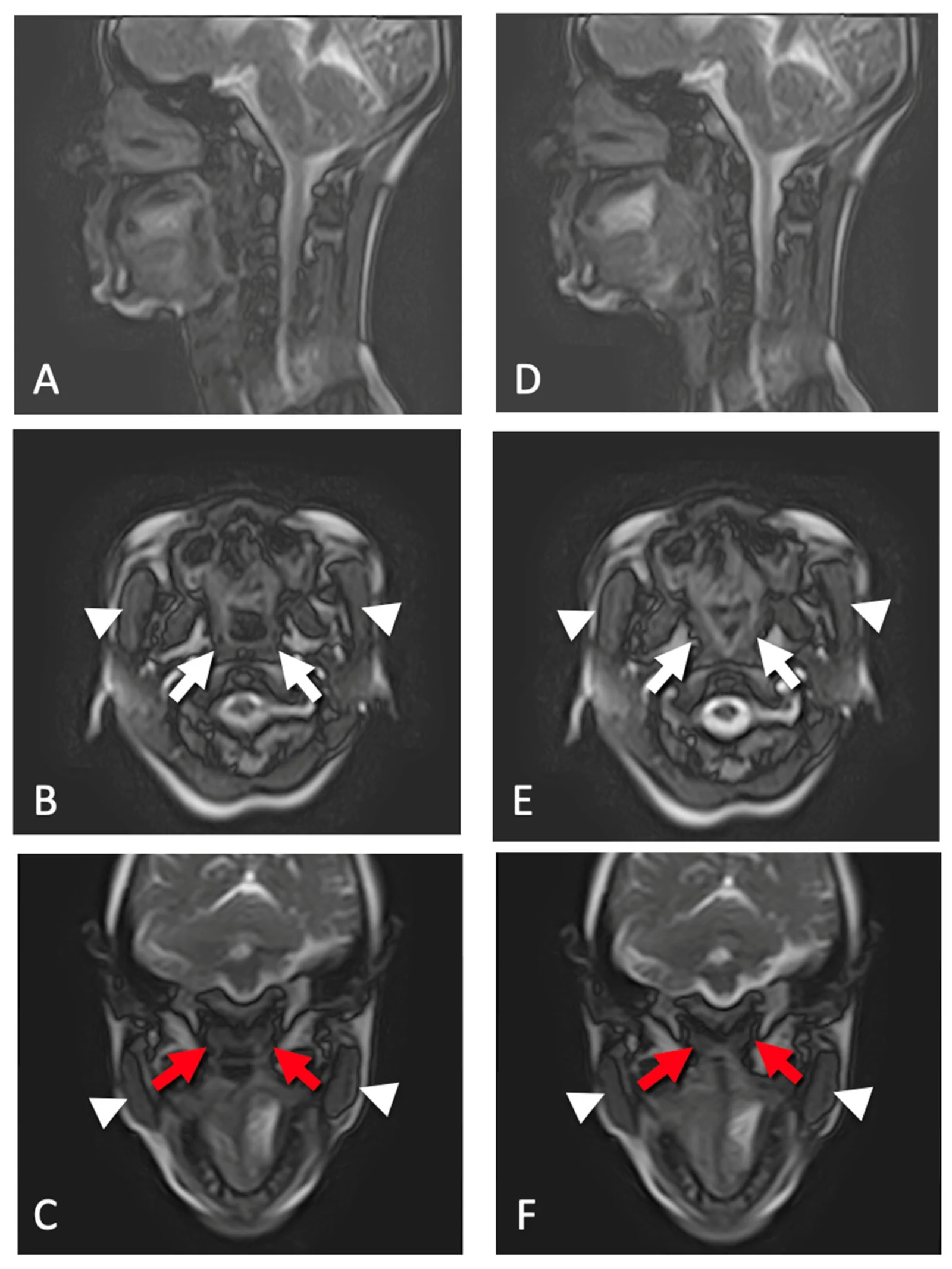
(**A**), Mid-sagittal; (**B**), axial; (**C**), and oblique coronal images of the velopharyngeal area during rest (1) and water swallowing (2) in the same patient as in Figure 2. During water swallowing, velopharyngeal closure is visualized, with the SP and LVPMs moving backward and contacting the posterior wall of the oropharynx anteriorly (**A**–**C**) and moving backward to contact the posterior wall of the oropharynx (**D**–**F**). In addition, the SPCMs expand anteriorly and bilaterally, resulting in posterior, right-, and left-sided closure (**D**–**F**). The signals of the SPCMs ((**B**,**E**); white arrows) and LVPMs ((**C**,**F**); red arrows) are conspicuously increased compared with rest. The signal intensities of the MMs ((**B**,**C**,**E**,**F**); arrowheads) show little change between rest and water swallowing.

The flow direction to the esophagus could be judged on post-operative CMR in all 19 patients (Figure 3, Video S2), but the flow direction to the trachea could not be judged. Structures important for swallowing, such as the anterior tongue, base of the tongue, SP, posterior pharyngeal wall, floor of the mouth, and epiglottis, could all be seen on CMR (Figure 3) and on video (Video S2). The mobility of the anterior tongue, base of the tongue, SP, floor of the mouth, and posterior pharyngeal wall could be accurately evaluated. The OTT, PTT, and tissue immobility score among the 12 CMR parameters deteriorated significantly between the pre- and post-operative assessments (Table 6). However, the SI_working_/SI_rest_ of the velopharyngeal-related muscles did not differ significantly between the pre- and post-operative assessments (Table 6).

Video S2. Video obtained during water swallowing shows the oral cavity, mid-velopharyngeal, and esophageal areas (mid-sagittal) in the same patient as in Figure 3. During water swallowing, the signals of the LVPMs and SPCMs are conspicuously increased compared with rest. The rate of increase in signal intensity of the SPCMs and LVPMs during water swallowing is marked. The signal intensities of the MMs show little change between rest and water swallowing. At the same time, velopharyngeal closure is visualized, with the SP and LVPMs moving backward to contact the posterior wall of the oropharynx. In addition, the SPCMs expand anteriorly and bilaterally, resulting in posterior, right-, and left-sided closure.

## 4. Discussion

In our recent studies, we demonstrated that our newly developed CMR technique using 1.5-T MRI systems is useful for evaluating swallowing and velopharyngeal functions [6,7,8,11]. The technique provides simple, safe, and non-invasive evaluation of these functions [6,7,8,11]. We also demonstrated that the MR signals of velopharyngeal-related muscles were markedly altered during water swallowing on CMR [11]. Therefore, we hypothesized that this approach could objectively evaluate the status of velopharyngeal-related muscles in patients with oral cancers and their response to treatment in a manner distinct from conventional methods.

The primary finding of this feasibility study is that the SI_working_/SI_rest_ ratio of the LVPMs and SCPMs may potentially provide an objective visualization of functional changes in patients with oral cancers, corresponding to contact between the SP and the posterior pharyngeal wall or movement of the SP. The change in the SI_working_/SI_rest_ of the LVPMs and SCPMs did not differ significantly during swallowing between the pre- and post-operative assessments, despite other CMR parameters indicating mild deterioration consistent with postoperative changes. However, the SI_working_/SI_rest_ ratio showed limited association with standard temporal swallowing parameters. This lack of correlation may suggest that the SI ratio in its present form is insensitive to minor swallowing alterations, or that localized muscle signal changes reflect internal contractile effort rather than global bolus transit efficiency.

To date, most cine-MRI studies evaluating swallowing function have used T1-weighted sequences [17,23,24]. To the best of our knowledge, there have been no reports of MR signal changes in the velopharyngeal-related muscles during swallowing except our previous study [11]. Therefore, the feasible present study provides the first objective criteria for evaluating patients with oral cancers and their treatments using signal changes in the velopharyngeal-related muscles on CMR. Regarding the physiological mechanism of T2-weighted signal enhancement during swallowing, blood flow increase during muscular contraction remains a plausible hypothesis, similar to dynamic changes observed in temporomandibular joint studies [25]. However, given the rapid dynamic nature of swallowing, non-physiological factors such as motion-related artifacts, tissue deformation, or partial volume effects cannot be ruled out and warrant further technical investigation.

In the feasible present study, the sequence of swallowing events observed was almost identical to that described in textbooks using VF with the individual upright [26]. In particular, the present technique allows the evaluation of swallowing function, in addition to visualization of three-dimensional defects and reconstructions without additional treatments. This technique is applied in clinical practice as a supplementary procedure to the MRI scans performed periodically after oral cancer surgery to assess recurrence and lymph node metastasis.

However, this feasible study has several significant methodological and clinical limitations that must be addressed: Firstly, the sample size was limited to 19 patients with heterogeneous primary sites, T-classifications, and surgical procedures. Consequently, subgroup analyses (Table 3, Table 4 and Table 5) were purely exploratory, lacked statistical power, and showed no statistically significant differences. Firm conclusions regarding specific surgical impacts cannot be drawn. Next, on patient cohort and disease severity, all enrolled patients had a relatively preserved swallowing function with normal self-reported scores. Because our facility is a dental university hospital, patients with severe dysphagia or severe velopharyngeal insufficiency were not included. Thus, the diagnostic utility of the SI ratio for detecting true pathological dysfunction remains unproven.

On the reproducibility and observer dependency, the image evaluations and ROI placements were performed by a single experienced oral radiologist to ensure internal consistency across our ongoing research series. However, inter-observer and intra-observer reliability were not formally tested. Cine-MRI interpretation and ROI placement may depend heavily on evaluator experience, and future studies comparing experienced and novice readers are necessary. In addition, single sagittal plane acquisitions were utilized due to scanner hardware constraints restricting simultaneous multi-planar dynamic imaging, and to maintain consistency with our past protocols.

Cine-MRI required patients to be in a supine position. This positional difference may skew parameters such as OTT and PTT. Additionally, the short acquisition window (approx. 10 s) risks missing delayed pharyngeal residue clearance or late aspiration events, which can take longer than 5 s in severe oral cancer cases. In this cohort, flow direction to the trachea could not be judged primarily because no patients exhibited clinical or overt aspiration during scanning. While T2-weighted sequence allows tracking of liquid boluses—meaning silent aspiration of fluids could theoretically be visualized—the inability to definitively rule out or evaluate tracheal penetration remains a major barrier before CMR can be considered a replacement for standard videofluorography (VFSS) or flexible endoscopic evaluation of swallowing (FEES). This study lacked direct simultaneous comparison with gold-standard modalities (VFSS/FEES), a healthy control group, and longitudinal long-term follow-up. Furthermore, clinical variables such as adjuvant radiotherapy/chemotherapy, medications, postoperative rehabilitation, and exact time elapsed since surgery were not sub-analyzed to avoid over-complicating this preliminary dataset.

## 5. Conclusions

The primary strength of this feasible study lies in introducing a safe, non-invasive, radiation-free CMR protocol that can be easily integrated into routine post-treatment MRI follow-ups without contrast media. Its main weakness is the small, underpowered, and non-symptomatic cohort, which limits the generalizability of our findings. Therefore, the current results should be interpreted cautiously as proof of concept. While CMR is a promising complementary tool, extensive external validation in larger, symptomatic patient populations against gold-standard techniques is required before broader clinical implementation.

## Figures and Tables

**Table 1:** Imaging parameters.

	Sequence		
	STIR	T1WI	Cine-MR
TR (ms)	4700	805	3.2
TE (ms)	75	10	1.6
Flip angle (°)	90	90	45
FOV (mm)	200 × 200	200 × 200	250 × 225
Section thickness (mm)	6	6	8
Matrix (pixels)	272 × 272	224 × 320	120 × 96

TR, repetition time; TE, echo time; FOV, field of view; STIR, short T1 inversion recovery; T1WI, T1-weighted image; Cine-MR, cine-magnetic resonance.

**Table 2:** Pre-operative SI ratios in velopharyngeal- and swallowing-related muscles.

	SI_rest_ (Mean ± SD)	SI_working_ (Mean ± SD)	SI Ratio (SI_working_/SI_rest_) (Mean ± SD)	Mean Difference (95% CI)	*p*-Value (Paired *t*-Test)	Q-Value (FDR)	Effect Size (Cohen’s dz)
SI							
OMs	999.04 ± 189.76	1037.26 ± 224.16	1.05 ± 0.15	38.2 (−36.5–112.9)	0.297	0.297	0.25
SP	1369.97 ± 544.77	1473.70 ± 450.47	1.16 ± 0.32	103.7 (−42.2–249.7)	0.153	0.184	0.34
MMs (oblique coronal)	856.56 ± 205.33	943.09 ± 286.97	1.10 ± 0.19	86.5 (0.2–172.8)	0.059	0.100	0.48
LVPMs	804.44 ± 221.61	1130.12 ± 234.08	1.45 ± 0.25	325.7 (244.5–406.9)	<0.001 *	<0.001 *	1.93
MMs (axial)	949.90 ± 178.91	1009.51 ± 182.51	1.08 ± 0.19	59.6 (−24.1–143.3)	0.152	0.184	0.34
SCPMs	1063.07 ± 188.05	2016.41 ± 515.27	1.93 ± 0.50	953.3 (706.6–1200.0)	<0.001 *	<0.001 *	1.86

* Statistically significant (*p*-value < 0.01, Q-value < 0.01). To adjust for multiple testing, q-values were calculated using the Benjamini–Hochberg false discovery rate (FDR) procedure. Effect sizes were reported as Cohen’s dz for paired samples. Confidence intervals (95% CI) represent the mean differences between resting and swallowing conditions. SI, signal intensity; CI, confidence interval; SD, standard deviation; OMs, occipital muscles; SP, soft palate; MMs, masseter muscles; LVPMs, levator veli palatini muscles; SCPMs, superior constrictor pharyngeal muscles.

**Table 3:** Correlation between pre-operative SI ratio of velopharyngeal-related muscles during water swallowing and T classification.

	Group					
	Tis/T1 (N = 8)	T2 (N = 4)	T3 (N = 2)	T4 (N = 5)	*p*-Value (One-Way ANOVA)	Effect Size (Partial η^2^)
SI ratio (SI_working_/SI_rest_)						
OMs	1.07 ± 0.10	0.90 ± 0.07	1.15 ± 0.24	0.93 ± 0.04	0.193	0.26
SP	1.21 ± 0.36	1.22 ± 0.39	1.24 ± 0.07	1.13 ± 0.17	0.686	0.09
MMs (oblique coronal)	1.06 ± 0.17	1.17 ± 0.29	1.02 ± 0.05	1.20 ± 0.10	0.720	0.08
LVPMs	1.46 ± 0.29	1.52 ± 0.26	1.21 ± 0.03	1.44 ± 0.04	0.604	0.11
MMs (axial)	1.17 ± 0.22	1.03 ± 0.10	0.88 ± 0.12	1.13 ± 0.05	0.242	0.24
SCPMs	1.94 ± 0.42	2.25 ± 0.45	1.58 ± 0.22	1.42 ± 0.10	0.464	0.15

Values are expressed as mean ± standard deviation. *p* values were calculated using one-way analysis of variance (ANOVA). There were no statistically significant differences among the groups for any of the muscles evaluated (all *p* > 0.05). Effect sizes were reported as Partial η^2^. Effect sizes for ANOVA were reported as partial eta squared (partial η^2^). SI, signal intensity; OMs, occipital muscles; SP, soft palate; MMs, masseter muscles; LVPMs, levator veli palatini muscles; SCPMs, superior constrictor pharyngeal muscles.

**Table 4:** Correlation between the SI ratio of velopharyngeal-related muscles during water swallowing and tumor site.

	Site			
	Tongue (n = 12)	Maxillary Gingiva (n = 1)	Mandibular Gingiva (n = 5)	Floor of the Mouth (n = 1)
SI ratio (SI_working_/SI_rest_)				
OMs	1.05 ± 0.16	1.12	0.96 ± 0.05	1.32
SP	1.13 ± 0.36	1.64	1.16 ± 0.20	0.99
MMs (oblique coronal)	1.12 ± 0.21	0.96	1.08 ± 0.16	1.14
LVPMs	1.50 ± 0.26	1.51	1.27 ± 0.17	1.71
MMs (axial)	1.10 ± 0.22	1.08	1.09 ± 0.06	0.83
SCPMs	1.99 ± 0.47	1.62	1.98 ± 0.58	1.34

SI, signal intensity; OMs, occipital muscles; SP, soft palate; MMs, masseter muscles; LVPMs, levator veli palatini muscles; SCPMs, superior constrictor pharyngeal muscles. Comparisons according to tumor site were presented descriptively because two tumor-site groups each contained only one patient, precluding meaningful statistical comparison.

**Table 5:** Correlation between post-operative SI ratio of velopharyngeal-related muscles and surgical procedure.

	Type I (n = 10)	Type IV (n = 9)	Mean Difference (95% CI)	*p*-Value (*T*-Test)	Effect Size (Cohen’s d)
SI ratio (SI_working_/SI_rest_)					
OMs	1.08 ± 0.09	1.04 ± 0.14	0.04 (−0.08–0.16)	0.518	0.30
SP	1.16 ± 0.35	1.20 ± 0.20	−0.04 (−0.33–0.25)	0.779	−0.13
MMs (oblique coronal)	1.03 ± 0.12	1.08 ± 0.18	−0.04 (−0.20–0.11)	0.569	−0.27
LVPMs	1.41 ± 0.14	1.55 ± 0.25	−0.14 (−0.35–0.07)	0.169	−0.66
MMs (axial)	1.05 ± 0.06	1.02 ± 0.14	0.03 (−0.08–0.13)	0.631	0.22
SCPMs	1.85 ± 0.40	1.64 ± 0.40	0.21 (−0.20–0.63)	0.297	0.49

Values are expressed as mean ± standard deviation. *p* values were calculated using Student’s *t*-test for independent samples. There were no statistically significant differences between Type I and Type IV surgical procedures for any of the muscles evaluated (all *p* > 0.05). Effect sizes were reported as Cohen’s d for samples. Confidence intervals (95% CI) represent the mean differences between type I and IV. Effect sizes were reported as Cohen’s d for independent samples. Confidence intervals (95% CI) represent the mean differences between Type I and Type IV surgical procedures. SI, signal intensity; OMs, occipital muscles; SP, soft palate; MMs, masseter muscles; LVPMs, levator veli palatini muscles; SCPMs, superior constrictor pharyngeal muscles.

**Table 6:** Correlation between pre- and post-operative SI ratios and CMR parameters of velopharyngeal-related muscles during water swallowing.

	Pre-Operative	Post-Operative	Mean Difference (95% CI)	*p*-Value (Paired *t*-Test)	Q-Value (FDR)	Effect Size (Cohen’s dz)
SI ratio (SI_working_/SI_rest_)						
Oms	1.05 ± 0.15	1.06 ± 0.12	0.01 (−0.06–0.08)	0.748	0.795	0.07
SP	1.16 ± 0.32	1.18 ± 0.29	0.02 (−0.17–0.21)	0.795	0.795	0.06
MMs (oblique coronal)	1.10 ± 0.19	1.05 ± 0.15	−0.05 (−0.13–0.03)	0.227	0.694	−0.29
LVPMs	1.45 ± 0.25	1.47 ± 0.21	0.02 (−0.13–0.18)	0.757	0.795	0.07
MMs (axial)	1.08 ± 0.19	1.04 ± 0.11	−0.04 (−0.16–0.07)	0.448	0.795	−0.18
SCPMs	1.93 ± 0.50	1.75 ± 0.42	−0.18 (−0.49–0.13)	0.231	0.694	−0.28
CMR parameter						
OTT (s)	1.21 ± 0.44	1.70 ± 0.90	0.49 (0.02–0.97)	0.044 *	0.241	0.5
PTT (s)	1.08 ± 0.37	1.31 ± 0.64	0.24 (0.02–0.45)	0.031 *	0.241	0.54
OOT (s)	1.15 ± 0.95	1.33 ± 0.79	0.18 (−0.06–0.43)	0.139	0.383	0.35
PR1 (s)	1.52 ± 1.02	1.63 ± 0.77	0.11 (−0.24–0.46)	0.506	0.695	0.16
LAT (s)	0.95 ± 0.40	1.17 ± 0.72	0.22 (−0.05–0.48)	0.101	0.372	0.4
GCT (s)	1.21 ± 0.64	1.14 ± 0.75	−0.08 (−0.29–0.14)	0.467	0.695	−0.17
Epiglottic retroflexion (s)	1.17 ± 0.66	1.09 ± 0.85	−0.09 (−0.32–0.15)	0.456	0.695	−0.17
Vallecular and piriform sinus filling (s)	0.91 ± 0.54	0.86 ± 0.47	−0.05 (−0.23–0.14)	0.605	0.739	−0.12
PR2 (s)	1.10 ± 1.28	1.07 ± 0.70	−0.04 (−0.36–0.29)	0.82	0.82	−0.05
EOT (s)	0.96 ± 0.42	0.99 ± 0.64	0.03 (−0.17–0.23)	0.74	0.814	0.08
LDT (s)	0.98 ± 0.39	1.12 ± 0.44	0.13 (−0.14–0.40)	0.32	0.695	0.23
Tissue immobility score	9.79 ± 0.95	10.00 ± 1.75	1.16 (0.23–2.08)	0.017 *	0.017	0.6

Among the time events: * statistically significant (*p*-value < 0.01, Q-value < 0.01). To adjust for multiple testing, q-values were calculated using the Benjamini–Hochberg false discovery rate (FDR) procedure. Effect sizes were reported as Cohen’s dz for paired samples. Confidence intervals (95% CI) represent the mean differences between pre- and post-operatives. SI, signal intensity; CMR, cine-magnetic resonance; OMs, occipital muscles; SP, soft palate; MMs, masseter muscles; LVPMs, levator veli palatini muscles; SCPMs, superior constrictor pharyngeal muscles; OTT, oral transit time; PTT, pharyngeal transit time; OOT, orovelar opening time; PR1, first Passavant ridge; LAT, laryngeal ascent time; GCT, glottal closure time; PR2, second Passavant ridge; EOT, esophageal opening time; LDT, laryngeal descent time.

## Data Availability

For confidentiality issues, the data will only be shared in aggregate form, as presented in the figures and tables.

## Supplementary Materials

The following supporting information can be downloaded at https://www.mdpi.com/article/10.3390/tomography12080117/s1. Video S1: Representative pre-operative cine-MRI. Video S2: Representative post-operative cine-MRI.
